# The Effect of Virtual Reality on Pain and Anxiety During Pap Smear Testing: A Randomized Controlled Trial

**DOI:** 10.3390/healthcare14213678

**Published:** 2026-10-30

**Authors:** Buse Nur Güney, Arzu Abiç

**Affiliations:** Department of Nursing, Faculty of Health Sciences, Eastern Mediterranean University, Famagusta 99628, Türkiye

**Keywords:** pap smear, virtual reality, pain management, anxiety

## Abstract

Introduction: Pap smear testing is an important cervical cancer screening procedure; however, the pain, discomfort, and anxiety experienced during the procedure may negatively affect women’s screening experiences and adherence to future screening. Virtual reality (VR) is a nonpharmacological intervention that may help manage procedure-related pain and anxiety through immersive distraction. This study aimed to evaluate the effect of VR on pain and anxiety during Pap smear testing. Methods: This randomized controlled experimental study included 60 women undergoing Pap smear testing. Participants were randomly assigned to an intervention group (*n* = 30) or a control group (*n* = 30). Data were collected using the Personal Information Form, the State-Trait Anxiety Inventory-State (STAI-S), and the Visual Analog Scale (VAS). During the Pap smear test procedure, the intervention group watched a relaxing nature video using a VR glasses, whereas the control group received routine nursing care. STAI-S was administered before and after the procedure, and pain intensity was assessed using the VAS after the procedure. Results: No significant difference was found between the groups in pretest STAI-S scores (39.83 ± 11.00 vs. 41.46 ± 10.10, *p* = 0.534). However, posttest STAI-S scores were significantly lower in the intervention group than in the control group (24.80 ± 6.70 vs. 40.13 ± 9.58, *p* < 0.001). Postprocedure VAS pain scores were also significantly lower in the intervention group compared with the control group (1.43 ± 1.25 vs. 2.86 ± 2.43, *p* = 0.028). Conclusions: Virtual reality was effective in reducing pain and anxiety during Pap smear testing. VR may be used as a simple, nonpharmacological nursing intervention to support pain and anxiety management and improve women’s comfort during gynecological procedures.

## 1. Introduction

Cervical cancer is one of the most common cancers affecting women worldwide and, despite being largely preventable, remains a major cause of morbidity and mortality [1,2,3]. According to World Health Organization data, approximately 660,000 women worldwide were diagnosed with cervical cancer in 2022, while 350,000 women died from the disease [2]. These data indicate that cervical cancer remains a serious global public health problem. The primary prevention of cervical cancer is possible through prophylactic HPV vaccination, while secondary prevention is achieved through regular screening programs. Bivalent and quadrivalent HPV vaccines can be administered to both females and males from the age of 9 years; the bivalent vaccine provides protection against HPV types 16 and 18, whereas the quadrivalent vaccine provides protection against HPV types 6, 11, 16, and 18. However, in Türkiye, the HPV vaccine is not included in the national vaccination schedule and is administered on a paid and voluntary basis [4]. The Papanicolaou (Pap) test, which is the most widely used method for secondary prevention, enables the early detection of abnormal cellular changes and is frequently preferred in screening programs because it is simple, rapid, and low-cost [5,6]. Cervical cancer screening strategies vary across countries and may include cytology-based screening, primary high-risk human papillomavirus (HPV) testing, or HPV/Pap co-testing. Primary HPV testing has increasingly been adopted as the preferred primary screening strategy in many countries because of its higher sensitivity for detecting clinically relevant precancerous lesions. However, cytology-based screening remains an important component of cervical cancer screening programs in several settings [7]. Nevertheless, despite the availability of screening methods, numerous factors hinder women’s participation in these services.

The greatest obstacle to the prevention of cervical cancer is women never undergoing screening [8]. Studies in the literature have identified numerous reasons why women do not undergo Pap smear testing. These include not considering cervical cancer a sufficiently serious disease, reluctance to discuss the subject, fear of experiencing pain, lack of knowledge about the importance of the test, time constraints, and fear of receiving a positive result, all of which have been reported to negatively affect participation in screening programs [9]. In addition, gynecological examinations can be an anxiety-provoking experience for women; inadequate attention to privacy and negative attitudes of healthcare personnel may further complicate this experience [10]. In the present study, anxiety refers specifically to state anxiety associated with the Pap smear procedure, rather than general or trait anxiety. State anxiety reflects a temporary emotional state that may fluctuate in response to a specific situation or procedure. Therefore, state anxiety was assessed immediately before and after the Pap smear procedure to evaluate changes associated with the intervention. In some cultural contexts, concerns related to privacy and modesty during gynecological examinations may delay women’s seeking healthcare services and increase their anxiety levels [11]. Studies have found that women who experience pain and discomfort during gynecological examinations have significantly higher anxiety scores [12]. This situation may negatively affect women’s participation in simple yet critically important screening methods such as the Pap smear testing, thereby reducing the opportunity for early diagnosis.

The frequent experience of fear, anxiety, and pain among women during gynecological examinations necessitates research into new methods that may facilitate this process [13]. The literature has demonstrated that cognitive–behavioral techniques used during short-term medical interventions reduce pain and anxiety by directing patients’ attention toward different stimuli [14]. Virtual reality (VR) technology, which has developed in recent years, has also emerged as a promising method in this context. VR applications, which provide users with a simulated alternative reality, have been reported to be effective in reducing pain and anxiety during medical procedures through their distracting effects [15,16]. Therefore, integrating VR technology into gynecological screening procedures has the potential to alleviate the negative experiences encountered by women.

Studies have found that the combined use of a virtual reality headset, distraction cards, and breathing exercises during Pap smear testing reduces women’s anxiety and pain levels while also increasing their satisfaction [16]. Similarly, evidence indicates that VR applications reduce pain, stress, and anxiety by distracting patients’ attention and increase satisfaction and quality of life [17]. However, studies on the use of VR technology in women’s health, particularly in screening procedures such as Pap smear testing, remain quite limited. This gap necessitates more comprehensive investigation of the subject.

In conclusion, it is important to investigate the effect of virtual reality technology applied during Pap smear testing on women’s pain and anxiety levels. The findings obtained are expected to provide an innovative perspective to nursing care, improve women’s adherence to gynecological examinations, and contribute to the quality of healthcare services. In this context, the primary aim of the study was to determine the effect of virtual reality application during Pap smear testing on women’s anxiety and pain levels.

Research Hypotheses:

**Hypothese 1.1.**  *There is a difference in pain levels between women in the intervention group who received the virtual reality glasses and those in the control group who did not receive the intervention.*

**Hypothese 1.2.**  *There is a difference in anxiety levels between women in the intervention group who received the virtual reality glasses and those in the control group who did not receive the intervention.*

## 2. Methods

### 2.1. Study Design and Setting

This study was conducted using a randomized controlled experimental design to evaluate the effectiveness of virtual reality (VR) as a nonpharmacological nursing intervention for the management of pain and anxiety during Pap smear testing. It was conducted following the guidelines of the Consolidated Standards of Reporting Trials (Supplementary Materials CONSORT 2025 Statement) [18,19]. The study was registered with ClinicalTrials.gov (NCT07462039) on 10 March 2026, and the registry URL has been provided. The registry URL was accessed on 28 October 2026: https://clinicaltrials.gov/study/NCT07462039. The study was conducted between April and June 2026 in the Obstetrics and Gynecology Outpatient Clinic of Life Hospital in Famagusta, Turkish Republic of Northern Cyprus. Life Hospital is a private hospital located in the district center and provides healthcare services across 20 specialties, including obstetric and gynecological care. Women presenting for Pap smear testing were initially evaluated by a physician in a consultation room. Following the initial assessment, participants were transferred to an examination room equipped with a gynecological examination chair and the medical equipment required for Pap smear testing. To minimize potential contamination between study groups, the waiting area was organized to limit direct interaction and visual contact between participants. Participants in the intervention and control groups were assessed at separate times, and the VR intervention was not demonstrated or described to participants in the control group.

### 2.2. Participants and Sample Size

Referring to the randomized controlled trial by Kurt and Özcan [20], the effect size for the difference in Visual Analog Scale (VAS) pain scores between the two groups was assumed to be Cohen’s d = 0.82. Using G*Power version 3.1.9.7, with a two-sided significance level of 0.05 and a statistical power of 80%, the sample size calculation indicated that a minimum of 52 participants (26 per group) was required. Considering a potential attrition rate of 10%, the target sample size was increased to 60 women (30 per group). Participants were randomly assigned in a 1:1 ratio to the virtual reality intervention group (*n* = 30) or the control group (*n* = 30).

### 2.3. Eligibility and Exclusion Criteria

Women were eligible to participate if they met all of the following criteria: were willing to participate in the study, were aged 18–65 years, were able to speak and read Turkish, and had no communication difficulties.

Women were excluded from the study if they met any of the following criteria: had a diagnosed psychiatric disorder, had hearing or visual impairment, or wore glasses, as the virtual reality glasses used in the study were not compatible with eyeglasses due to the design of the device. Participants were withdrawn from the study if they removed the virtual reality glasses before completion of the Pap smear testing or failed to complete the postprocedure questionnaires or submitted incomplete forms.

### 2.4. Study Variables and Outcome Measures

The independent variable was the VR intervention administered during Pap smear testing. The primary outcomes were pain intensity and state anxiety following the Pap smear testing procedure. Pain intensity was assessed using the Visual Analog Scale (VAS), and state anxiety was assessed using the State Anxiety Inventory (STAI-S).

### 2.5. Data Collection Instruments

Data were collected using a Personal Information Form, the STAI-S, and the VAS.

### 2.6. Personal Information Form

The Personal Information Form was developed by the researchers based on a review of the literature and consisted of 14 questions. Six questions addressed sociodemographic characteristics, including age, educational status, employment status, income status, and previous use of a VR glasses. Eight questions assessed obstetric characteristics [16,17,21].

### 2.7. State Anxiety Inventory

The State Anxiety Inventory (STAI-S) was developed by Spielberger et al. [22] and adapted into Turkish by Öner and Le Compte [23]. The instrument consists of 20 items rated on a four-point Likert scale (1 = not at all, 2 = somewhat, 3 = moderately, 4 = very much). Items 1, 2, 5, 8, 10, 11, 15, 16, 19, and 20 are reverse scored. Total scores range from 20 to 80, with higher scores indicating greater anxiety. Scores of 36 or below indicate no anxiety, scores of 37–42 indicate mild anxiety, and scores of 43 or above indicate high anxiety. These categories were based on the Turkish adaptation and validation of the STAI-S and were used descriptively rather than as universally applicable clinical diagnostic cutoffs. The internal consistency coefficient reported by Öner and Le Compte [23] ranged from 0.94 to 0.96. In the present study, Cronbach’s alpha was 0.79. The STAI-S was administered and scored according to the Turkish version, including the specified reverse-coded items.

### 2.8. Visual Analog Scale for Pain

Pain intensity was assessed using a 10 cm Visual Analog Scale (VAS). The scale ranged from 0 cm, indicating “no pain,” to 10 cm, indicating “unbearable pain.” Participants marked the point corresponding to their perceived pain intensity. Higher scores represented greater pain intensity. The VAS is a simple and commonly used measure of pain intensity and is sensitive to changes associated with pharmacological and nonpharmacological pain management interventions [24].

### 2.9. Randomization and Allocation Concealment

A computer-generated randomization sequence was prepared by an independent researcher, and 60 sequentially numbered, opaque, sealed envelopes were prepared according to the allocation sequence. Eligible participants were recruited according to their examination order. After the participant was admitted to the examination room, provided written informed consent, and completed the baseline assessments, the corresponding envelope was opened by the researcher immediately before the Pap smear testing procedure to determine the group assignment. The researcher then administered the intervention according to the assigned group.

**Figure 1:**
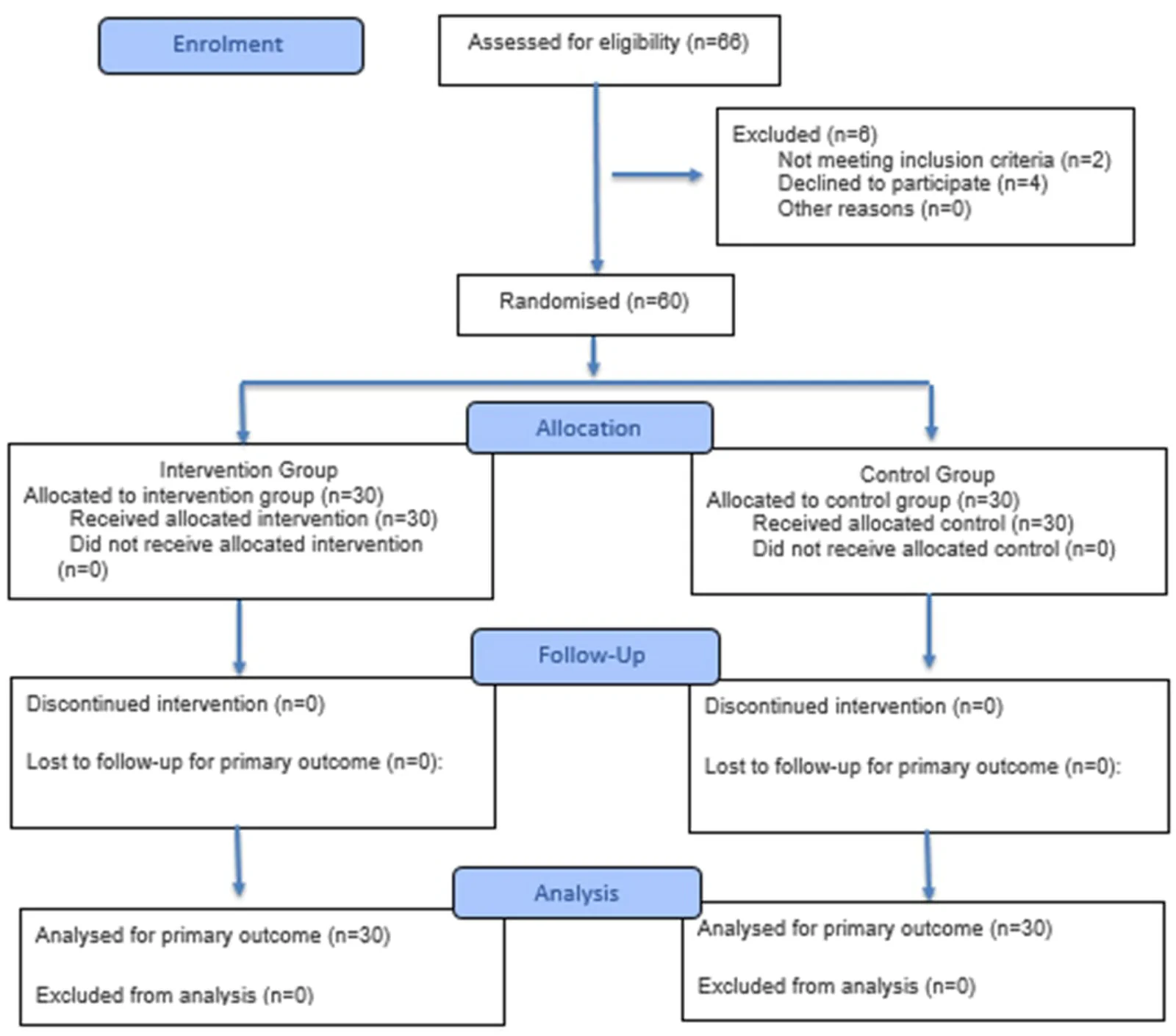
CONSORT (Consolidated Standards of Reporting Trials) flow diagram of participants through the study.

Due to the nature of the intervention, participants and the researcher who adminisered the intervention could not be blinded to group allocation. However, the data were coded by group numbers before statistical analysis, and the statistical analysis was performed using coded data without revealing group identities to the analyst. To minimize the risk of contamination, participants did not encounter or interact with participants from the other group, as each participant was assessed individually in the examination room and group allocation was revealed only after entering the examination room. A total of 66 women were assessed for eligibility. Of these, six were excluded: two because they were older than 65 years and did not meet the predefined age criterion, and four because they declined to participate. The remaining 60 eligible women were enrolled in the study and randomly assigned to the intervention group (*n* = 30) or the control group (*n* = 30). (Figure 1). All 60 randomized participants were analyzed in the groups to which they were originally assigned. No participants were replaced after randomization, and no primary outcome data were missing.

### 2.10. Intervention and Data Collection Procedure

Before the Pap smear testing procedure, all potential participants who met the predefined eligibility criteria completed the Personal Information Form and the STAI-S. The STAI-S was administered before the procedure to assess participants’ baseline state anxiety levels. Participants who met all eligibility criteria and agreed to participate were enrolled in the study. Following completion of the Pap smear testing procedure, participants returned to the waiting area after dressing and completed the postprocedure STAI-S and VAS approximately 10 min after the procedure. The pain and anxiety scales were administered approximately 10 min after the Pap smear procedure to assess the pain and anxiety experienced during the procedure. The assessments were conducted after the procedure to avoid disrupting the immersive environment and participants’ focus during the VR intervention.

### 2.11. Intervention Group

The VR intervention was designed as a nonpharmacological distraction-based pain and anxiety management intervention. Before the procedure, participants were introduced to the virtual reality glasses and informed that they would wear them throughout the Pap smear testing procedure. Before the procedure, participants were provided with routine procedural information and reassurance regarding the examination. The researcher remained with the participant throughout the procedure and provided routine support as needed. After participants were positioned on the gynecological examination chair, the virtual reality glasses (Pico G2 4K, Model A7510) connected to Bose SoundLink II AE headphones were applied. No video was shown during the introduction. After participants were positioned on the gynecological examination chair, the virtual reality glasses were applied. The instruments required for the Pap smear testing procedure were prepared behind the examination table and positioned so that they were not directly visible to the participant, consistent with the routine clinical practice applied during the examination. During the entire Pap smear testing procedure, participants watched the same 5 min, high-resolution, relaxing 360° nature video through the virtual reality glasses. The video featured green natural scenery and a flowing stream, accompanied by the sound of flowing water (https://m.youtube.com/watch?v=6ud18oSuCiY&t=42s&pp=2AEqkAIB&ra=m, accessed on 20 October 2026). The same video was used for all participants in the intervention group to ensure standardization of the intervention. The intervention was intended to provide immersive distraction from the clinical environment, procedural stimuli, and medical instruments. The Pap smear testing was completed by the clinician according to the hospital’s standard clinical procedure. Following completion of the procedure, the virtual reality glasses were removed, and participants were allowed to dress in privacy. They subsequently completed the postprocedure STAI-S and VAS in the waiting area. The postprocedure assessment took approximately 10 min. The virtual reality glasses were disinfected after each use. The postprocedure STAI-S and VAS assessments were conducted approximately 10 min after completion of the Pap smear testing procedure, in the waiting area. This interval was selected to allow sufficient time for participants to leave the examination chair, dress in privacy, and return to the waiting area, while maintaining a standardized and short interval between the procedure and postprocedure assessment. Thus, the immediate effects of the intervention on pain and state anxiety could be assessed while minimizing the influence of unrelated factors that might occur with a longer delay.

### 2.12. Control Group

Participants assigned to the control group received routine nursing care during the Pap smear testing procedure and did not receive VR or any additional distraction intervention. They completed the Personal Information Form and STAI-S before the procedure. Participants were then positioned on the gynecological examination chair and prepared for the Pap smear testing procedure according to the hospital’s routine clinical practice and standard procedure. Before the procedure, participants were provided with routine procedural information and reassurance regarding the examination. The researcher remained with the participant throughout the procedure and provided routine support as needed. The instruments required for the procedure were prepared behind the examination table and kept out of the participant’s direct view, consistent with the routine clinical practice applied during the examination. No additional intervention or distraction technique was provided beyond the hospital’s routine procedure. After the procedure, participants were allowed to dress in privacy and subsequently completed the postprocedure STAI-S and VAS. The assessment process took approximately 10 min. The postprocedure STAI-S and VAS assessments were conducted approximately 10 min after completion of the Pap smear testing procedure, in the waiting area. This interval was selected to allow sufficient time for participants to leave the examination chair, dress in privacy, and return to the waiting area, while maintaining a standardized and short interval between the procedure and postprocedure assessment. Thus, the immediate effects of the intervention on pain and state anxiety could be assessed while minimizing the influence of unrelated factors that might occur with a longer delay.

### 2.13. Statistical Analysis

Data were analyzed using IBM SPSS Statistics for Windows, Version 26.0 (IBM Corp., Armonk, NY, USA). Descriptive statistics were reported as frequencies, percentages, means, standard deviations, minimums, and maximums. Distributional assumptions were assessed using the One-Sample Kolmogorov–Smirnov test. Because STAI-S scores did not demonstrate a normal distribution, nonparametric tests were used. Pain intensity and state anxiety were considered co-primary outcomes, assessed using the Visual Analog Scale (VAS) and the State-Trait Anxiety Inventory-State (STAI-S), respectively. No hierarchical order was prespecified between the two primary outcomes. The outcomes were evaluated separately, and evidence of efficacy was not contingent on statistically significant effects on both outcomes. No adjustment for multiple comparisons was prespecified. The intervention and control groups were compared regarding categorical sociodemographic and obstetric characteristics using the chi-square test or Fisher’s exact test, as appropriate. The Mann–Whitney U test was used for comparisons of continuous variables between independent groups. Within-group changes in pretest and posttest STAI-S scores were evaluated using the Wilcoxon signed-rank test. Between-group comparisons of posttest anxiety and pain scores were performed using the Mann–Whitney U test. The internal consistency of the STAI-S was assessed using Cronbach’s alpha. Statistical significance was established at *p* < 0.05, with all tests conducted as two-sided tests.

Effect sizes were calculated to quantify the magnitude of the observed differences. For between-group comparisons using the Mann–Whitney U test, approximate Cohen’s d values were derived from the standardized Z statistics using the effect size conversion method provided by Psychometrica (https://www.psychometrica.de/effect_size.html, accessed on 20 October 2026). For within-group comparisons using the Wilcoxon signed-rank test, the effect size r was calculated using the formula r = Z/N, where Z represents the standardized test statistic and N represents the number of paired observations included in the analysis. Hodges–Lehmann estimates and their 95% confidence intervals were reported to describe the magnitude, direction, and precision of between-group differences.

During the revision process, an additional sensitivity analysis was performed to further evaluate the between-group difference in changes in State Anxiety Inventory scores. Individual change scores were calculated by subtracting posttest scores from pretest scores, with positive values indicating a reduction in anxiety. Because the change scores did not meet the assumption of normality, the intervention and control groups were compared using the Mann–Whitney U test. The between-group difference was further described using the Hodges–Lehmann estimate and its 95% confidence interval. An approximate Cohen’s d effect size was calculated by converting the Z statistic from the Mann–Whitney U test. This additional analysis was not included in the original analysis plan and was conducted during the revision process to assess the robustness of the findings regarding anxiety reduction. The results of this supplementary analysis are presented separately in from those of the originally planned analyses. A separate statistical analysis plan was not prepared for this study. The statistical methods used are described in detail in this section.

### 2.14. Ethical Considerations

The study was conducted in accordance with the ethical principles of the Declaration of Helsinki. Ethical approval was obtained from the Eastern Mediterranean University Scientific Research and Publication Ethics Board, Health Ethics Subcommittee, on 17 November 2025 (Approval No. ETK00-2025-22806). Institutional permission was obtained from Life Hospital. All participants provided written informed consent after receiving information regarding the purpose and procedures of the study. Permission to use the STAI-S was obtained from the relevant author via e-mail.

## 3. Results

A total of 60 women were included in the study, with 30 participants in the intervention group (*n* = 30) and 30 in the control group (*n* = 30). In the intervention group, 73.3% of the women had a bachelor’s degree or higher, 73.3% were employed, 76.7% were married, and 60.0% reported that their income was equal to their expenses. Among the women, 83.3% had not previously used a virtual reality headset, 86.7% had previously undergone a Pap smear testing, and 83.3% of those who had undergone a Pap smear testing had performed so as part of routine screening. In the intervention group, 40.0% of the women had two previous births, 43.3% used withdrawal as a contraceptive method, and 56.7% had no family history of gynecological cancer. In addition, 46.7% of the women had not previously received information or education about the Pap smear testing, while 43.3% of those who had received information or education reported that their source of information was a physician.

In the control group, 66.7% of the women had a bachelor’s degree or higher, 73.3% were employed, 66.7% were married, and 50.0% reported that their income was equal to their expenses. Among the women, 90.0% had not previously used a virtual reality headset, 90.0% had previously undergone a Pap smear testing, and 93.3% of those who had undergone the test had performed so as part of routine screening. In the control group, 36.7% of the women had two previous births, 36.7% used withdrawal as a contraceptive method, and 83.3% had no family history of gynecological cancer. Furthermore, 56.7% of the women had not previously received information or education about the Pap smear testing, while 33.3% of those who had received information or education reported that their source of information was a physician.

No statistically significant differences were found between the intervention and control groups in terms of age, education level, employment status, marital status, previous use of a virtual reality headset, previous Pap smear testing and reason for testing, number and mode of delivery, contraceptive method, previous information or education about the Pap smear testing, and source of information (*p* > 0.05). In contrast, a statistically significant difference was found between the groups in terms of family history of gynecological cancer (*p* < 0.05) (Table 1).

In Table 2, the State Anxiety Inventory scores of the intervention and control groups were compared. In the intervention group, the mean scale score decreased from 39.83 at pretest to 24.80 at posttest, whereas the corresponding scores in the control group were 41.46 and 40.13, respectively. No statistically significant difference was found between the groups’ pretest scores (*p* = 0.534), whereas the difference between their posttest scores was statistically significant (*p* < 0.001). A significant decrease was observed between pretest and posttest scores in the intervention group (*p* < 0.001), while no significant change was found in the control group (*p* = 0.451).

In Table 3, the State Anxiety Inventory scores did not differ significantly between the intervention and control groups at pretest (U = 408.00, *p* = 0.534). At posttest, the intervention group had significantly lower anxiety scores than the control group (U = 84.00, *p* < 0.001). The between-group difference was 15.00 points (95% CI: 12.00–20.00), with a large effect size (Cohen’s d = 1.953). Within-group analysis showed a significant reduction in anxiety scores in the intervention group (Z = −4.24, *p* < 0.001; η^2^ = 0.547), whereas no significant change was observed in the control group (Z = −0.754, *p* = 0.451). The 95% confidence interval for the pretest–posttest change was −18.50 to −11.00 in the intervention group and −3.00 to 1.00 in the control group (Table 3).

In Table 4, as an additional analysis conducted during the revision process, pretest–posttest change scores in State Anxiety Inventory scores were compared between the intervention and control groups using the Mann–Whitney U test. A statistically significant difference was found between the groups (U = 114.00, Z = −4.97, *p* < 0.001). The mean change score was 15.03 ± 12.33 in the intervention group and 1.33 ± 6.70 in the control group. The mean ranks were 41.70 for the intervention group and 19.30 for the control group. The Hodges–Lehmann estimate of the between-group difference was −14.00 (95% CI: −18.00 to −10.00). The approximate Cohen’s d effect size, converted from the Z statistic of the Mann–Whitney U test, was 1.672, indicating a large effect. These findings provide additional evidence of a greater reduction in anxiety scores in the intervention group.

Table 5, the mean posttest Visual Analog Scale (VAS) pain score was lower in the intervention group (1.43 ± 1.25) than in the control group (2.86 ± 2.43). The difference between the groups was statistically significant (Mann–Whitney U = 304.50, *p* = 0.028). The Hodges–Lehmann estimate of the between-group difference was 1.00 points (95% CI: 0.00–2.00), with positive values indicating higher pain scores in the control group. The approximate effect size derived from the Mann–Whitney test was Cohen’s d = 0.578, suggesting a moderate effect. These findings indicate that participants who received the virtual reality intervention reported lower pain scores during Pap smear testing than those receiving routine care.

## 4. Discussion

Precancerous changes in the cervix can be detected at an early stage by Pap smear testing before progressing to cancer. Pap smear testing is an effective screening method that is easy to perform, low-cost, reliable, and widely applicable in population-based screening programs [25]. Anxiety and pain experienced by women during Pap smear collection are important factors affecting cervical cancer screening behavior. In particular, fear of receiving a cancer diagnosis, the perception that the procedure will be painful, feelings of embarrassment, and inadequate information before the test may increase women’s anxiety levels. In addition, the approach of healthcare professionals, perceptions of privacy, and the gender of the person performing the procedure may also influence women’s anxiety [26]. Various nonpharmacological methods are used to reduce anxiety and improve women’s Pap smear testing experience [27]. Virtual reality, one of these methods, is among the effective approaches that can reduce anxiety and pain perception by diverting individuals’ attention away from the procedure [28]. In this study, the effects of virtual reality applied during Pap smear collection on women’s anxiety and pain were examined, and the findings were evaluated in accordance with the literature.

When the sociodemographic and obstetric characteristics of women in the intervention and control groups were compared, no statistically significant differences were observed between the groups for most variables (*p* > 0.05). However, the absence of statistically significant differences does not necessarily indicate baseline equivalence between the groups. A statistically significant difference was observed in family history of gynecological cancer (*p* = 0.024) (Table 1). In addition, although the majority of women had previously undergone Pap smear testing, the proportion of women who had received information or education about the test was relatively low. This finding highlights the need to strengthen education and counseling services regarding cervical cancer screening. According to the findings of the study, there was no statistically significant difference in state anxiety levels between the intervention and control groups before the procedure (*p* > 0.05). After the procedure, however, a significant decrease in state anxiety scores was observed in the intervention group, whereas this decrease was not pronounced in the control group (Table 2). These results indicate that virtual reality was effective in reducing women’s state anxiety levels during Pap smear testing. The additional sensitivity analysis comparing anxiety change scores between the intervention and control groups further supported the observed difference in anxiety reduction. The mean reduction in anxiety scores was greater in the intervention group than in the control group (15.03 ± 12.33 vs. 1.33 ± 6.70), and the between-group comparison was statistically significant (U = 114.00, Z = −4.971, *p* < 0.001). These findings provide additional evidence of a greater reduction in anxiety scores in the intervention group. Nevertheless, this analysis compared change scores and did not adjust for potential baseline imbalances in other participant characteristics; therefore, the findings should be interpreted in conjunction with the study’s limitations. These findings confirm our hypothesis (H1.2) that there is a difference in anxiety levels between women in the intervention group who received the virtual reality headset and those in the control group who did not receive the intervention. The reduction in STAI-S scores from 39.83 to 24.80 in the intervention group represents a substantial decrease in state anxiety and may indicate a meaningful improvement in women’s psychological experience during Pap smear testing. However, because a predefined minimal clinically important difference (MCID) was not established in this study, the clinical significance of this change should be interpreted cautiously. The literature indicates that virtual reality (VR) applications are used as an effective nonpharmacological method for reducing anxiety during invasive and semi-invasive procedures. Keleş and Toker [17] examined the effect of virtual reality on women’s anxiety levels. In their study, the preprocedural anxiety levels of the intervention and control groups were similar, whereas after the procedure, anxiety levels significantly decreased in the intervention group receiving virtual reality, while no significant change was observed in the control group. These findings are consistent with the results of our study and support that virtual reality is an effective method for reducing anxiety before and during procedures in women [17]. These findings are also supported by the results of a meta-analysis by Van Haaps et al. [29], which included 13 randomized controlled trials. The meta-analysis evaluated the effects of virtual reality on pain and anxiety during various gynecological procedures, such as outpatient hysteroscopy, intrauterine device (IUD) insertion, postpartum episiotomy repair, colposcopy, and hysterosalpingography (HSG). In the meta-analysis, which included a total of 1107 women, pain and anxiety were generally reduced in groups receiving virtual reality; subgroup analyses showed that preprocedural anxiety was significantly reduced particularly in diagnostic gynecological procedures, while postprocedural anxiety was significantly reduced in combined procedures [29]. These results are consistent with the significant reduction in postprocedural state anxiety levels observed in the intervention group in our study and support that virtual reality is an effective nonpharmacological method for anxiety management during gynecological procedures. The decrease in anxiety levels observed in our study is thought to be associated with the ability of virtual reality to divert individuals’ attention away from the clinical environment and procedure-related negative stimuli, as well as to provide users with a relaxing virtual environment different from the real environment, thereby reducing the perception of environmental stimuli. Virtual reality applications enable individuals to focus intensely on the virtual environment, suppressing cognitive processes related to pain and anxiety, increasing the sense of relaxation, and thus contributing to a more positive perception of the procedural experience [30]. The effects of VR on pain and anxiety may involve mechanisms beyond simple distraction. Immersive VR can modulate attention by directing cognitive resources toward the virtual environment and away from procedure-related stimuli, thereby reducing the cognitive processing of pain and anxiety. In addition, according to gate control theory, cognitive and emotional factors can influence the transmission and perception of pain. By reducing attention to painful stimuli and promoting relaxation, VR may contribute to the modulation of pain perception. These mechanisms may help explain the reductions in pain and anxiety observed in the present study. However, contrary to our study, Hecken et al. [31] found no statistically significant effect of virtual reality on anxiety during colposcopy [31]. This difference may be attributed to the fact that colposcopy is a more invasive procedure than Pap smear testing, takes longer, and may cause greater discomfort. In addition, differences in participant characteristics, the virtual reality content used, intervention duration, timing of the intervention, and measurement methods between studies may also explain the differences in the findings. In conclusion, the findings of the current study are consistent with a substantial proportion of the studies in the literature and support that virtual reality can be used as an effective and nonpharmacological intervention to reduce women’s anxiety during Pap smear testing. Accordingly, integrating virtual reality applications into routine nursing practices to support anxiety management in women’s health services may be beneficial. However, the implementation of VR should not necessarily be considered simple in all clinical settings. Access to appropriate equipment, staff training, device cleaning and maintenance, cost, and integration into the clinical workflow may present practical barriers to routine implementation. Therefore, the feasibility of VR should be considered in relation to available resources and clinical settings.

The lower mean pain scores among women in the intervention group compared with the control group suggest that virtual reality may help reduce perceived pain during Pap smear testing (Table 3). This finding may be explained by the ability of virtual reality to divert individuals’ attention away from the procedure and thereby reduce pain perception. Although the between-group difference was statistically significant at the nominal level, no multiplicity adjustment was prespecified for the two primary outcomes. Therefore, the finding should be interpreted cautiously and does not provide definitive evidence that the pain hypothesis (H1.1) was confirmed. In the study conducted by Kurt and Özcan [20], post-pelvic examination pain scores were significantly lower among women in the intervention group than in the control group. This finding is consistent with the results of the present study and suggests that virtual reality may help reduce perceived pain during gynecological examinations [20]. Similarly, a study examining the effect of virtual reality on pain among women after cesarean section reported significantly lower post-intervention pain scores in the intervention group than in the control group (*p* < 0.001). The reduction in pain scores within the intervention group was also statistically significant (*p* < 0.001). These findings provide further support for investigating virtual reality as a potential nonpharmacological approach to pain management [32]. In conclusion, when the findings and the literature are considered together, virtual reality appears to be an effective and potentially useful nonpharmacological method for pain management. Particularly during short-term gynecological procedures such as Pap smear testing, virtual reality diverts individuals’ attention away from the procedure, reduces perceived anxiety and pain, and makes the procedural process more tolerable. However, it should be considered that the effectiveness of the method may vary depending on factors such as the content used, individual characteristics, and duration of the intervention. Overall, virtual reality applications are considered an important supportive approach for reducing anxiety and pain. Patient preference should also be considered when implementing VR in clinical practice, as not all women may feel comfortable with or prefer using VR during a gynecological procedure. In addition, the cost-effectiveness of VR was not evaluated in the present study. Future research should consider equipment, training, maintenance, and implementation costs together with patient acceptability and clinical benefits to determine whether VR represents a cost-effective approach for Pap smear testing. This study has some limitations. The generalizability of the findings is limited because the study was conducted at a single center, the sample was restricted to a specific region, and only the short-term effects during the procedure were evaluated. In addition, long-term patient satisfaction and repeat screening behavior were not assessed.

This study is important because it evaluates the effects of virtual reality as a nonpharmacological pain management intervention during Pap smear testing on women’s pain and anxiety levels together. In this respect, it makes an important contribution to the limited literature on the use of virtual reality in gynecological procedures. The findings of the study indicate that virtual reality may be an effective nonpharmacological method for reducing women’s anxiety and pain levels during Pap smear testing and support its consideration as a feasible approach to improving patient comfort during gynecological procedures. Therefore, studies conducted with larger samples and different populations to investigate the integration of virtual reality applications into clinical practice are considered likely to contribute to the literature.

## 5. Conclusions

### Study Limitations

The study has several limitations. It was conducted in a single hospital with a relatively small sample of 60 women, which may limit the generalizability of the findings. Most participants had previous Pap smear experience, which may limit the applicability of the findings to women undergoing Pap smear testing for the first time. Women who wore glasses were excluded because the VR device used in the study was not compatible with regular eyeglasses. This exclusion may limit the applicability of the findings to women who routinely wear glasses. Only the immediate effects of VR on pain and state anxiety were assessed; longer-term outcomes such as psychological effects, satisfaction, and subsequent Pap smear testing attendance were not evaluated. Pain and anxiety were assessed using self-report instruments and may therefore have been influenced by individual perceptions and emotional states. Individual differences in previous VR experience, familiarity with technology, and attentional responses could not be fully controlled and may have influenced the intervention outcomes. Another limitation is that the sample size calculation was based on an effect size reported in a previous study and was not calculated separately for pain and anxiety outcomes. Therefore, the study may have had different statistical power for these outcomes. A further limitation is that adjustments for multiple comparisons were not performed. Consequently, the risk of type I error may have increased due to the number of statistical tests conducted. Although the analyses were based on the predefined study outcomes, the findings should therefore be interpreted cautiously, particularly for secondary and exploratory comparisons, and warrant confirmation in future studies with appropriate multiplicity adjustment. The clinician and outcome assessor were not blinded, which may have introduced bias. The self-reported pain and anxiety outcomes may have also been influenced by this lack of blinding. The study compared the overall VR intervention package with routine care; therefore, the effects of individual components could not be distinguished. No adverse effects or other discomforts were reported by the participants during the study. However, as adverse effects were not systematically assessed, conclusions regarding the safety of the intervention should be drawn with caution.

## Figures and Tables

**Table 1:** Sociodemographic and obstetric characteristics of the participants in the intervention and control groups.

Variables	Intervention Group (*n*: 30)		Control Group (*n*: 30)		Statistical Test *	
	*n*	%	*n*	%	χ^2^	*p*
Educational Status					4.97	0.290 ^2^
Primary school	1	3.3	1	3.3		
Middle school	2	6.7	0.0	0.0		
High school	5	16.7	9	30.0		
Bachelor’s degree	11	36.7	14	46.7		
Master’s degree or higher	11	36.7	6	20.0		
Employment Status					0.000	1.000 ^1^
Employed	22	73.3	22	73.3		
Unemployed	8	26.7	8	26.7		
Marital Status					0.739	0.390 ^1^
Married	23	76.7	20	66.7		
Single	7	23.3	10	33.3		
Income Status					0.732	0.694 ^1^
Income less than expenses	4	13.3	6	20.0		
Income equal to expenses	18	60.0	15	50.0		
Income greater than expenses	8	26.7	9	30.0		
Previous Use of a Virtual Reality Headset					0.577	0.448 ^1^
Yes	5	16.7	3	10.0		
No	25	83.3	27	90.0		
Previous Pap Smear Testing					0.162	0.688 ^1^
Yes	26	86.7	27	90.0		
No	4	13.3	3	10.0		
Reason for Undergoing Pap Smear Testing					1.97	0.373 ^1^
Routine screening	25	83.3	28	93.3		
Abnormal discharge	4	13.3	1	3.3		
Abnormal bleeding	1	3.3	1	3.3		
Number of Births					1.35	0.852 ^2^
No previous births	11	36.7	11	36.7		
One birth	4	13.3	5	16.7		
Two births	12	40.0	11	36.7		
Three births	2	6.7	3	10.0		
Four births	1	3.3	0.0	0.0		
Mode of Delivery					0.381	0.944 ^1^
No previous births	11	36.7	11	36.7		
Vaginal delivery	7	23.3	7	23.3		
Cesarean delivery	10	33.3	11	36.7		
Both vaginal and cesarean delivery	2	6.7	1	3.3		
Contraceptive Method Used					1.78	0.775 ^1^
None	5	16.7	7	23.3		
Condom	8	26.7	6	20.0		
Intrauterine device	1	3.3	3	10.0		
Oral contraceptive pill	3	10.0	3	10.0		
Withdrawal	13	43.3	11	36.7		
Family History of Gynecological Cancer					5.07	0.024
Yes	13	43.3	5	16.7		
No	17	56.7	25	83.3		
Previous Information or Education About the Pap Smear Testing					0.601	0.438 ^1^
Yes	16	53.3	13	43.3		
No	14	46.7	17	56.7		
Source of Information or Education About the Pap Smear Testing					4.01	0.404 ^2^
No education received	14	46.7	17	56.7		
Physician	13	43.3	10	33.3		
Midwife	0.0	0.0	1	3.3		
Nurse	2	6.7	0.0	0.0		
Social environment	1	3.3	2	6.7		
Age	Mean	SD	Mean	SD	U: 392.0	0.391
	39.06	11.47	41.43	11.58		

* Statistical Test Used = ^1^: Cross-tabulation and Chi-square Test, ^2^: Fisher’s Exact Test. Abbreviations = Mean: Arithmetic Mean, and SD: Standard Deviation.

**Table 2:** Comparison of state anxiety inventory scores between the intervention and control groups.

Scale	Intervention Group (*n*: 30)		95% CI		Control Group (*n*: 30)		95% CI		Statistical Analysis *	Cohen’s d
	Mean ± SD	Median (IQR)			Mean ± SD	Median (IQR)				
State Anxiety Inventory–Pretest	39.83 ± 11.0	39.00 (15.50)	35.72	−43.94	41.46 ± 10.10	40.00 (14.50)	37.69	−45.24	U: 408.00–*p* = 0.534	0.161
State Anxiety Inventory–Posttest	24.80 ± 6.70	22.00 (8.00)	22.29	−27.30	40.13 ± 9.58	38.50 (15.25)	36.55	−43.71	U: 84.00–*p* < 0.001	1.953
Statistical Analysis **	Z: −4.24–*p* < 0.001		η^2^: 0.547		Z: −0.754–*p* = 0.451		η^2^: 0.097			

Statistical techniques used: * U = Mann–Whitney U test; ** Z = Wilcoxon signed-rank test. Abbreviations: Mean: Arithmetic Mean; SD: Standard Deviation.

**Table 3:** Comparison of state anxiety inventory scores between the intervention and control groups, including between-group effect estimates and 95% confidence intervals.

Scale and Subscales	Intervention Group (*n*: 30)		Control Group (*n*: 30)		Statistical Analysis *	95% CI	Cohen’s d
	Mean ± SD	Median (IQR)	Mean ± SD	Median (IQR)			
State Anxiety Inventory–Pretest	39.83 ± 11.0	39.00 (15.50)	41.46 ± 10.10	40.00 (14.50)	U: 408.00 *p*: 0.534	−3.00–7.00	---
State Anxiety Inventory–Posttest	24.80 ± 6.70	22.00 (8.00)	40.13 ± 9.58	38.50 (15.25)	U: 84.00 *p* < 0.000	12.00–20.00	1.953 *
Statistical Analysis **	Z: −4.24–*p* < 0.000 η^2^: 0.547 *		Z: −0.754–*p*: 0.451 η^2^: ---				
	**95% CI**		**95% CI**				
Lower Bound	−18.50		−3.00				
Upper Bound	−11.00		1.00				

Cohen’s d was calculated as an approximate effect size converted from the Z statistic of the Mann–Whitney U test. https://www.psychometrica.de/effect_size.html (accessed on 20 October 2026). * Between-group comparisons were performed using the Mann–Whitney U test. Cohen’s d indicates the effect size. ** Within-group pretest–posttest comparisons were performed using the Wilcoxon signed-rank test. η^2^ indicates the effect size.

**Table 4:** Additional between-group comparison of pretest–posttest change scores in state anxiety inventory scores.

	Intervention Group (*n*: 30)		Control Group (*n*: 30)		Statistical Analysis	95% CI		Hodges–Lehmann Estimate	Cohen’s d
	Mean ± SD	Median (IQR)	Mean ± SD	Median (IQR)					
Change Score	15.03 ± 12.33	15.00 (12.00)	1.33 ± 6.70	0.500 (6.00)	U: 114.00 *p* < 0.000	−18.00	−10.00	−14.00	1.672 *

* Between-group comparisons of change scores were performed using the Mann–Whitney U test. Cohen’s d indicates the effect size.

**Table 5:** Comparison of visual analog pain scale scores between the intervention and control groups.

Scale	Intervention Group (*n*: 30)			Control Group (*n*: 30)			Statistical Test	Cohen’s d
	Mean ± SD	Median (IQR)	95% CI	Mean ± SD	Median (IQR)	95% CI		
Visual Analog Pain Scale	1.43 ± 1.25	1.50 (2.00)	0.966–1.90	2.86 ± 2.43	2.00 (3.25)	0.00–3.77	U: 304.50– *p* = 0.028	0.578

Statistical technique used: U = Mann–Whitney U test. Abbreviations: Mean: Arithmetic Mean; SD: Standard Deviation. https://www.psychometrica.de/effect_size.html(accessed on 20 October 2026).

## Data Availability

The data and analysis code supporting the findings of this study are not publicly available due to privacy and ethical considerations. However, they are available from the corresponding author upon reasonable request.

## Supplementary Materials

The following supporting information can be downloaded at: https://www.mdpi.com/article/10.3390/healthcare14213678/s1. The CONSORT reporting checklist.
